# Prevalence of High Aldosterone-Renin Ratio in Patients With Hypertension in Basrah

**DOI:** 10.7759/cureus.36085

**Published:** 2023-03-13

**Authors:** Zeid Zwain, Hussein A Nwayyir, Haider A Alidrisi, Abbas A Mansour

**Affiliations:** 1 Diabetes and Endocrinology, Faiha Specialized Diabetes, Endocrine and Metabolism Center (FDEMC) University of Basrah, Basrah, IRQ; 2 Medicine, University of Basrah College of Medicine, Basrah, IRQ; 3 Diabetes and Endocrinology, University of Basrah College of Medicine, Basrah, IRQ

**Keywords:** iraq, basrah, aldosteronism, hypertension, aldosterone-renin ratio

## Abstract

Background and aim: Aldosterone-renin ratio (ARR) is an important screening tool for the assessment of primary aldosteronism as a cause of secondary hypertension. This study aimed to measure the prevalence of patients with elevated ARR among samples of Iraqi patients with hypertension.

Methods: A retrospective study was conducted at Faiha Specialized Diabetes, Endocrine and Metabolism Center (FDEMC) in Basrah during the period of February 2020 to November 2021. We evaluated the records of patients with hypertension whom were screened for endocrine cause, and an ARR cut-off equal or more than 5.7 was considered elevated.

Results: Of the total 150 patients enrolled, 39 (26%) of them had an elevated ARR. No statistically significant association for the elevated ARR with age, gender, BMI, duration of hypertension, systolic and diastolic blood pressure, pulse rate, and presence or absence of diabetes mellitus or lipid profile.

Conclusion: High frequency of elevated ARR was seen in 26% of patients with hypertension. More studies need to be conducted in the future taking larger samples.

## Introduction

Primary aldosteronism (PA) is defined as non-suppressible aldosterone secretion causing hypertension, which was regarded as the most common cause of secondary (endocrine) hypertension [1-4]. The classic type of PA showed hypokalemia associated with hypertension, but this form is seldom seen recently [5,6]. PA was first described by Conn in 1956 [7]. The importance of PA lies behind many points like it may be a reversible cause of hypertension and complications of hypertension and that increased aldosterone production prone the person to cardiovascular and renal morbidity and mortality in addition to increased risk of metabolic syndrome and diabetes which can be prevented earlier if PA diagnosed earlier [8-11]. Accordingly, many authorities developed screening criteria for PA. The most common one is the Endocrine Society guidelines in 2016 that stated eight groups of hypertensive patients should be screened for PA by measuring plasma aldosterone-renin ratio (ARR) [6].

Prevalence of elevated ARR varies from region to region, ranging from 1% to 30% in different pieces of literature and this depends on the population being studied, degree of blood pressure elevation, prospective or retrospective data, and type of test used [12-14]. In the Framingham offspring study, 12% of participants with untreated hypertension had elevated ARR [15], while in two german epidemiological studies, up to 7% of hypertensive participants had elevated ARR [16]. Torino's study in Italy used screening ARR and further confirmatory tests and found a prevalence of PA ranging from 3.9% in those with stage I hypertension to 11.8% in those with stage III hypertension [17]. In Middle East, ARR was poorly studied; we found a single study from Turkey-Trabzon where ARR was elevated at 17.4% in a random hypertensive cohort [18]. The present study aimed to determine the frequency of elevated ARR among a sample of Iraqi patients with hypertension in Basrah.

## Materials and methods

Study design

A retrospective study was conducted at Faiha Specialized Diabetes, Endocrine and Metabolism Center (FDEMC) in Basrah. We collected data registered from the period of February 2020 to November 2021. 

Data collection

The collected data include recorded ARR of patients with hypertension, and a cut-off equal to or more than 5.7 was considered elevated. One hundred fifty patients with hypertension were enrolled in this study. The other parameters retrieved were age, sex, body mass index (BMI), systolic and diastolic blood pressure (BP), pulse rate at the time of ARR measurement, duration of hypertension, creatinine, potassium level, and random lipid panel. Venous blood was drone while patients were seated for 15 minutes and not fasting.

Inclusion and exclusion criteria

The inclusion criteria included patients with age between 3 and 18 years, both male and female, and patients with hypertension fulfilling the 2016 Endocrine Society criteria for screening of PA [6]. The exclusion criteria included the patients with chronic kidney disease (estimated glomerular filtration rate {eGFR} < 60mL/min, CKD-EPI Equation 2021), mineralocorticoid receptor antagonists used in the last six weeks, and hyperkalemia (serum potassium > 5mEq/L). Other anti-hypertensive medications in the form of beta blockers, angiotensin-converting enzyme inhibitors (ACE inhibitors), angiotensin receptor blockers (ARBs), calcium channel blockers (CCBs), and diuretics were continued to avoid the risks associated with their discontinuations.

Biochemical analysis

Direct renin concentration (DRC) measured by Renin ELISA (REF EIA-5125; DRG Internation, Inc.: Springfield, NJ) (reference range: 7.54-42.3 ng/L). Plasma aldosterone concentration (PAC) measured by Aldosterone ELISA (REF KDO053; DiaMetra: Spello, Italy) (reference range: 1.3-23.1 ng/dL). The ARR is equal to PAC/DRC with a cut-off of ARR < 5.7 considered normal [6]. Serum potassium was measured using COBAS INTEGRA 400 plus (reference range: 3.5-5 mEq/L).

Statistical analysis

The data was analyzed using Statistical Package for Social Sciences version 28.0 for (Chicago, IL: SPSS Inc.) as follows: continuous variables were summarized as mean value ± standard deviation and categorical variables were summarized as percentages and frequencies. Chi-square (X²) test was used to study the correlation between categorical variables, while the independent Student's t-test was used to study the correlation between continuous variables and categorical variables. We adopt the two-tailed probability values with p≤0.05 to be statistically significant.

## Results

The general characteristics of the study patients in form of age, gender, BMI, presence or absence of diabetes, and duration of hypertension are summarized in Table 1.

**Table 1:** General characteristics of the patients screened for ARR (n=150) BMI, body mass index; SD, standard deviation; ACE, angiotensin-converting enzyme; ARBs, angiotensin receptor blockers; CCBs, calcium channel blockers

Age (years)	Mean ± SD	50.4±13.1
	≥50, n (%)	74 (49.3%)
	<50, n (%)	76 (50.7%)
Gender	Male, n (%)	78 (52.0%)
	Female, n (%)	72 (48.0%)
BMI (kg/m^2^)	Mean ± SD	32.2±5.3
	Obese (BMI ≥ 30), n (%)	95 (63.8%)
	Non-obese (BMI < 30), n (%)	54 (36.2%)
Diabetes duration of hypertension (years)	No, n (%)	89 (59.3%)
	Yes, n (%)	61 (40.7%)
	Range	1-43
Anti-hypertensive drugs	Beta-blockers, n (%)	34 (22.7%)
	ACE inhibitors, ARBs n (%)	54 (36%)
	CCBs, n (%)	31 (20.7%)
	Diuretics, n (%)	29 (19.3%)

In Table 2 and on the basis of the study cut-off for AAR, 111 (74%) patients had normal AAR and 39 (26%) had elevated ARR. There were no significant differences between these groups in terms of age, gender, BMI, systolic and diastolic blood pressure, pulse rate, potassium, cholesterol, triglyceride, low-density lipoprotein (LDL), very low-density lipoprotein (VLDL), non-high-density lipoprotein (HDL) cholesterol, HDL and presence or absence of diabetes (p > 0.05). The currently used anti-hypertensive medications in the form of beta blockers, ACE inhibitors, ARBs, and CCBs did not affect the prevalence of high AAR. But within patients on diuretics, the prevalence of high AAR was 10.3% as compared to 29.8% in patients without current diuretics use (p = 0.032).

**Table 2:** Variables associated with elevated ARR (n=150) K^+^, potassium; BP, blood pressure; LDL, low-density lipoprotein; VLDL, very low-density lipoprotein; HDL, high-density lipoprotein; ARR, aldosterone-renin ratio; ACE, angiotensin-converting enzyme; ARBs, angiotensin receptor blockers; CCBs, calcium channel blockers

Variable		Normal ARR (n= 111)	Elevated ARR (n= 39)	p-Value
Age (years)		49.4±12.7	53.2±13.6	0.118
Male		57 (73%)	21 (27%)	0.788
Female		54 (75%)	18 (25%)	
BMI		31.9 ±5.2	32.8 ±5.7	0.372
Systolic BP (mmHg)		154.6±22.1	160.9±26.9	0.152
Diastolic BP (mmHg)		95.3±11.5	95.5±13.2	0.930
Pulse rate (beat/min)		86.2±14.3	82.2±14.1	0.144
K^+^ (mEq/L)		4.3±0.4	4.4±0.5	0.123
Cholesterol (mg/dL)		195.6±42.9	190.4±45.0	0.535
Triglyceride (mg/dL)		217.6±119.3	255.9±189.0	0.161
LDL (mg/dL)		126.5±38.2	115.8±41.6	0.164
VLDL (mg/dL)		43.5±23.8	51.1±37.8	0.161
Non-HDL cholesterol (mg/dL)		153.6±41.2	149.8±46.5	0.652
HDL (mg/dL)		41.6±10.0	40.3±11.4	0.516
Diabetes	Yes	41 (67%)	20 (33%)	0.117
	No	70 (79%)	19 (21%)	
Beta-blockers	Yes	22 (64.7%)	12 (35.3%)	0.160
	No	89 (76.7%)	27 (23.3%)	
ACE inhibitors, ARBs	Yes	40 (74.1%)	14 (25.9%)	0.988
	No	71 (74%)	25 (26%)	
CCBs	Yes	23 (74.2%)	8 (25.8%)	0.978
	No	88 (73.9%)	31 (26.1%)	
Diuretics	Yes	26 (89.7%)	3 (10.3%)	0.032
	No	85 (70.2%)	36 (29.8%)	

## Discussion

This study looked at the frequency of elevated ARR among a cohort of Iraqi patients with hypertension matching screening criteria for secondary endocrine hypertension namely primary aldosteronism. FDEMC is a tertiary care clinic, and hence the screened hypertensive population had a high pretest probability of having elevated ARR. We found that 26% of the studied patients had elevated ARR, and those patients with elevated ARR must then undergo confirmatory tests to establish or exclude PA to avoid future cardiovascular risks and to optimize their blood pressure control. 

In comparison with this study, Algün et al. in Turkey conducted screening for elevated ARR in a referring tertiary center and found that ARR was elevated in 17.4% of the studied patients, which is nearly similar results to our study findings despite that Algün et al. conducted more confirmatory tests to confirm the diagnosis of PA [18], while Gilani et al. in Pakistan found lower total number of elevated ARR of 10% where his study was conducted also at a referring center [19]. This reflects that the total number of elevated ARR is different among different population groups taking into consideration the cut-off in each study and the population studied and the cofounders.

This was supported by studies across Europe where Hannemann et al. in Germany found that the total number of elevated ARR was 7% in population-based study [16]. While in the Torino study, a common study in Italy that was done at multiple primary care clinics, 21.1% of patients were found to have elevated ARR despite both studies being conducted in Europe [17].

The current study found a poor correlation between diabetes and elevated ARR, a similar result to Loh et al. despite they used confirmed PA and found no relation between the presence or absence of diabetes [3]. In comparison, Hanslik et al. found increased frequency of confirmed PA among patients with diabetes, but they used confirmatory tests to diagnose PA rather than elevated ARR screening done in current study [10].

Other parameters in this study, such as sex, age, lipid profile, systolic and diastolic BP, pulse rate, and duration of hypertension, were all studied and were found to be statistically non-significant and this may be due to the small sample size and the study not powered to study these parameters.

Limitations

First, the study sample showed high prevalence of elevated ARR because of referral center bias and single center. Furthermore, the center received all difficult hypertension cases from Basrah, Iraq, for evaluation of ARR. Second, the current use of anti-hypertensive medications might over or underestimate the ARR value. 

## Conclusions

Elevated ARR is common in Iraqi patients with hypertension (26% of patients) matching screening criteria. This could not reflect the true frequency of high ARR ratio due to study limitations. More studies need to be conducted in future including larger samples and collecting records from both tertiary centers and primary care centers.
